# Cryogenic electron microscopy reveals morphologically distinct subtypes of extracellular vesicles among porcine ejaculate fractions

**DOI:** 10.1038/s41598-024-67229-w

**Published:** 2024-07-13

**Authors:** Ana Parra, Isabel Barranco, Pablo Martínez-Díaz, Esperanza González, Oihane E. Albóniga, Diana Cabrera, Juan M. Falcón-Pérez, Jordi Roca

**Affiliations:** 1 Department of Medicine and Animal Surgery, Veterinary Science, University of Murcia, Murcia, Spain; 2 Exosomes Laboratory, Center for Cooperative Research in Biosciences (CIC bioGUNE), Basque Research and Technology Alliance (BRTA), Derio, Vizcaya Spain; 3 Centro de Investigación Biomédica en Red de Enfermedades Hepáticas y Digestivas, Madrid, Spain; 4 Metabolomics Platform, Center for Cooperative Research in Biosciences, Basque Research and Technology Alliance, Derio, Spain; 5 IKERBASQUE, Basque Foundation for Science, Bilbao, Spain

**Keywords:** Cryo-electron microscopy, Ejaculate fractions, Extracellular vesicles, Seminal plasma, Porcine, Reproductive biology, Cryoelectron microscopy

## Abstract

Seminal plasma (SP) is rich in extracellular vesicles (EVs), which are still poorly studied, especially in livestock species. To better understand their functional role in both spermatozoa and endometrial epithelial cells, proper characterization of EVs is an essential step. The objective was to phenotypically characterize porcine seminal EVs (sEVs) using cryogenic electron microscopy (cryo-EM), which allows visualization of EVs in their native state. Porcine ejaculates are released in fractions, each containing SP from different source. This allows characterization sEVs released from various male reproductive tissues. Two experiments were performed, the first with SP from the entire ejaculate (n:6) and the second with SP from three ejaculate fractions (n:15): the first 10 mL of the sperm-rich ejaculate fraction (SRF-P1) with SP mainly from the epididymis, the remainder of the SRF (SRF-P2) with SP mainly from the prostate, and the post-SRF with SP mainly from the seminal vesicles. The sEVs were isolated by size exclusion chromatography and 1840 cryo-EM sEV images were acquired using a Jeol-JEM-2200FS/CR-EM. The size, electron density, complexity, and peripheral corona layer were measured in each sEV using the ImageJ software. The first experiment showed that sEVs were structurally and morphologically heterogeneous, although most (83.1%) were small (less than 200 nm), rounded, and poorly electrodense, and some have a peripheral coronal layer. There were also larger sEVs (16.9%) that were irregularly shaped, more electrodense, and few with a peripheral coronal layer. The second experiment showed that small sEVs were more common in SRF-P1 and SRF-P2, indicating that they originated mainly from the epididymis and prostate. Large sEVs were more abundant in post-SRF, indicating that they originated mainly from seminal vesicles. Porcine sEVs are structurally and morphologically heterogeneous. This would be explained by the diversity of reproductive organs of origin.

## Introduction

Seminal plasma (SP), the fluid secreted in the male reproductive tract that surrounds spermatozoa during and after ejaculation, is rich in active biomolecules such as proteins, nucleic acids and metabolites that play key roles in regulating sperm functionality and the uterine environment^1^. In fact, SP biomolecules are involved in essential sperm functions such as motility, capacitation, and acrosome reaction^2^. Moreover, SP promotes a favorable uterine immune environment for sperm transit and embryo development and implantation once delivered to the female genital tract during mating or artificial insemination (AI)^3^.

In addition to active biomolecules, SP also contains many extracellular vesicles (EVs), which are nanoparticles surrounded by a lipid bilayer that carry active biomolecules and play a key role in cell-to-cell communication^4^. In fact, SP contains comparatively more EVs than other relevant body fluids such as blood or cerebrospinal fluid^5^. Despite this, EVs in SP (sEVs) are still less studied than those that circulate in other body fluids. A recent survey showed that less than 1% of EV research studies have been conducted on sEV^6^. Therefore, phenotypic characterization and elucidation of the functional roles of sEVs remains an important area of research. This is particularly evident in livestock species, where even fewer studies have been conducted^7^. Moreover, the few existing studies evaluating the involvement of sEVs in the functional performance of sperm and endometrial epithelial cells have reported inconsistent or even contradictory results^7^. One of the factors proposed to explain the inconsistent or contradictory functional results of sEVs would be their phenotypic and compositional heterogeneity^8,9^. The population of EV present in body fluids is phenotypically heterogeneous^10^, specially in SP, as sEVs originate from multiple sources, namely testis, vas deferens, epididymis, and accessory sex glands^7^. Therefore, it is imperative to identify and characterize the diversity of EV subtypes present in SP to better understanding the functional roles of sEVs.

Phenotypic characterization of EVs is an essential and mandatory first step to address any functional study of EVs and performing it with an orthogonal approach is crucial to provide solid evidence that the isolated particles are indeed EVs^11^. Several techniques are currently available to phenotypically characterize EVs, with those that allow the analysis of single EV, such as cryogenic electron microscopy (cryo-EM), gaining prominence^12–15^. In addition to its ability to analyze individual EVs, cryo-EM is the optimal imaging technique for the assessment of EV morphology and size. It allows the visualization of EVs in their native state, as it does not require dehydration, and provides high spatial resolution, allowing detailed analysis of EV structure^16,17^. Currently, there are studies focusing on the characterization of EVs from body fluids by cryo-EM^15,18–21^, but to our knowledge, only two of the sEVs are known to exist, both in the human species^22,23^. Furthermore, the study by Höög and Lötvall does not focus specifically on sEVs, but rather on all nanoparticles that are present in the SP^23^. Therefore, studies characterizing sEVs by cryo-EM remain of scientific interest, particularly in species other than humans. In addition, cryo-EM is an excellent technique for identifying and characterizing the different subtypes of EVs present in SP because it allows the analysis of individual EVs^11^. Moreover, cryo-EM is an optimal technique for phenotypic characterization of the peripheral coronal layer surrounding some EVs^24^, which has functional relevance and is another important phenotypic feature for defining EV subtypes^25^.

The pig is a globally important livestock species and a relevant animal model for human reproductive health^26^. Porcine ejaculate, like human ejaculate^27^, is expelled in two major fractions, with the SP of each fraction having a different origin. Specifically, SP in the first fraction, the sperm-rich fraction (SRF), originates primarily from the epididymis and prostate, and SP in the second fraction, the sperm-poor fraction (post-SRF), originates primarily from the seminal vesicles^28^. In addition, the first 10 mL of SRF (SRF-P1), which contains almost all the epididymal fluid present in the SP, can be collected separately from the remainder of the SRF (SRF-P2)^28^. In this context, the aim of the present study was to phenotypically characterize porcine sEVs by cryo-EM, including those of specific ejaculate fractions: SRF-P1, SRF-P2 and post-SRF. This may allow to determine whether sEV subtypes can be associated with specific EV-secreting reproductive organs.

## Results

### Complementary phenotypic characterization of sEV

Phenotypic characteristics of sEV-enriched samples from entire ejaculate samples showed that the total protein concentration was 359.5 ± 83.6 μg/mL. The mean particle size was 146 ± 26 nm (85 and 165 nm for the 25% and 75% percentiles, respectively) as determined by dynamic light scattering (DLS) (Supplementary Fig. S1A). Flow cytometry analysis showed that most particles analyzed (78.4% ± 6.2%) were positive for carboxyfluorescein succinimidyl ester (CFSE) and would therefore be considered sEVs. The mean concentration of sEVs was 1.5 × 10^11^ ± 0.2 × 10^11^ sEVs/mL. The majority of sEVs expressed the EV-specific protein markers CD63 (79.2% ± 2.9%) and HSP90β (71.9% ± 6.5%). Flow cytometry analysis also showed a low percentage of albumin-positive particles (5.5% ± 1.6%), indicating that the sEV samples were minimally contaminated by non-vesicular extracellular particles (NVEPs). Representative flow cytometry images are shown in Supplementary Fig. S1B and C.

Phenotypic characteristics of sEV-enriched samples from the three ejaculate fractions showed that the total protein concentration was higher (P < 0.05) in SRF-P1 samples (278.2 ± 65.6 μg/mL) than in post-SRF samples (186.6 ± 31.5 μg/mL), with intermediate values in the SRF-P2 (222.7 ± 52.5 μg/mL) (Supplementary Fig. S2A). The sEV concentration, measured by flow cytometry as CFSE-positive events, differed among the three ejaculate fractions (P < 0.001), with SRF-P1 and post-SRF samples having the higher (2.2 × 10^11^ ± 0.4 × 10^11^ sEVs/mL) and lower (1.2 × 10^11^ ± 0.2 × 10^11^ sEVs/mL) concentrations, respectively (Supplementary Fig. S2B). DLS revealed that the particle size was larger (P < 0.01) in the post-SRF samples (90 and 180 nm for the 25% and 75% percentiles, respectively) than in the SRF-P1 and SRF-P2 samples, the two sperm-rich ejaculate fractions (80 and 145 nm for the 25% and 75% percentiles, respectively) (Supplementary Fig. S2C). The percentages of sEVs expressing CD63 and HSP90β were high and similar in all three ejaculate fractions. Specifically, CD63-positive sEVs were 77.3% ± 4.8%, 78.6% ± 3.0%, and 74.2% ± 5.2% for SRF-P1, SRF-P2, and post-SRF, respectively. HSP90β positive sEVs were 82.5% ± 6.1%, 81.6% ± 4.8%, and 84.4% ± 6.1% for SRF-P1, SRF-P2, and post-SRF, respectively. The sEV samples from all three ejaculate fractions had low percentages of albumin-positive particles. However, the percentages in the post-SRF (6.41% ± 1.44%) were higher (P < 0.05) than in the SRF-P1 (3.47% ± 1.22%) and the SRF-P2 (3.96% ± 1.35%).

### Cryo-EM characterization of sEV of entire ejaculates

**Figure 1:**
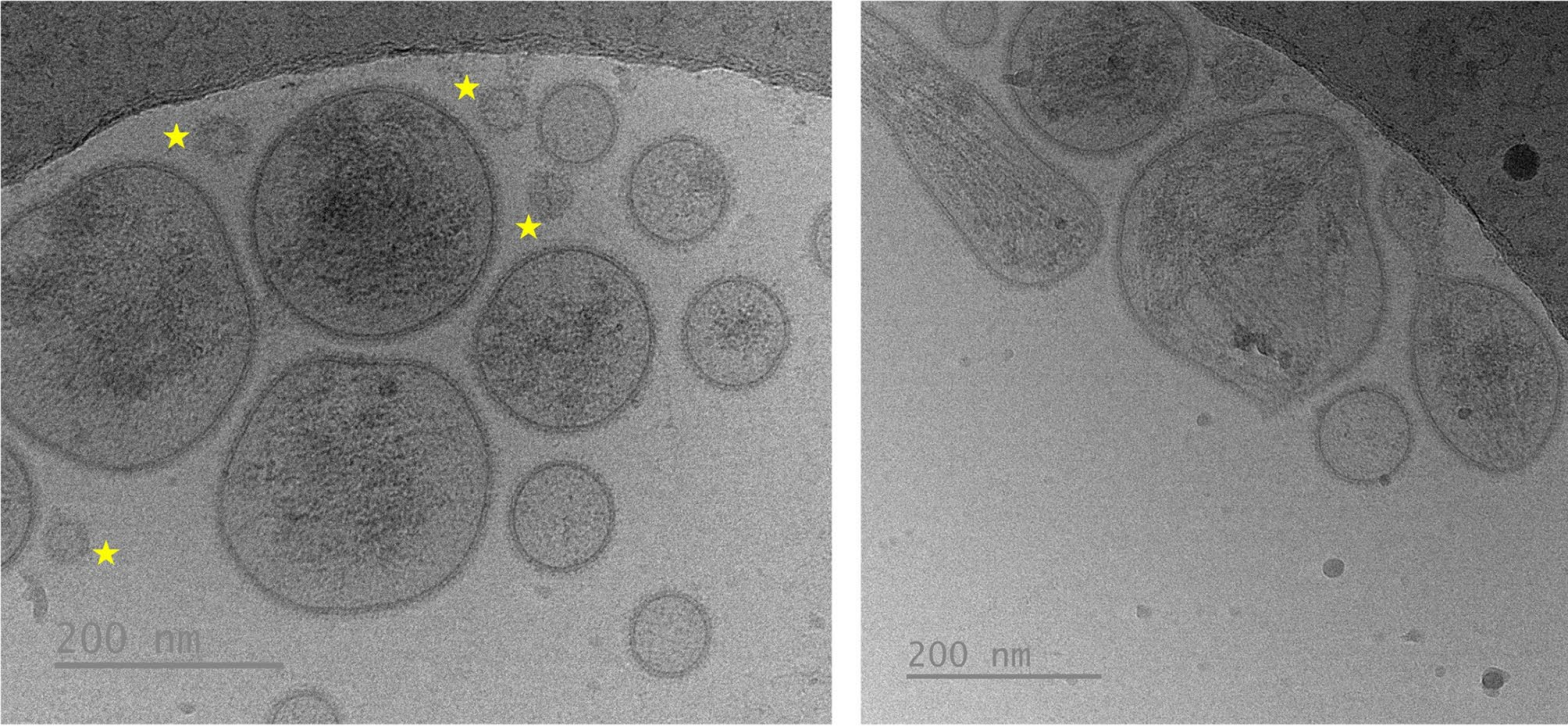
Representative cryo-electron microscopy images showing the structural diversity of seminal extracellular vesicles (sEVs) isolated from seminal plasma of entire porcine ejaculates. The yellow star identifies non-vesicular extracellular particles, which are similar in size to small sEVs but are not surrounded by a membrane.

**Figure 2:**
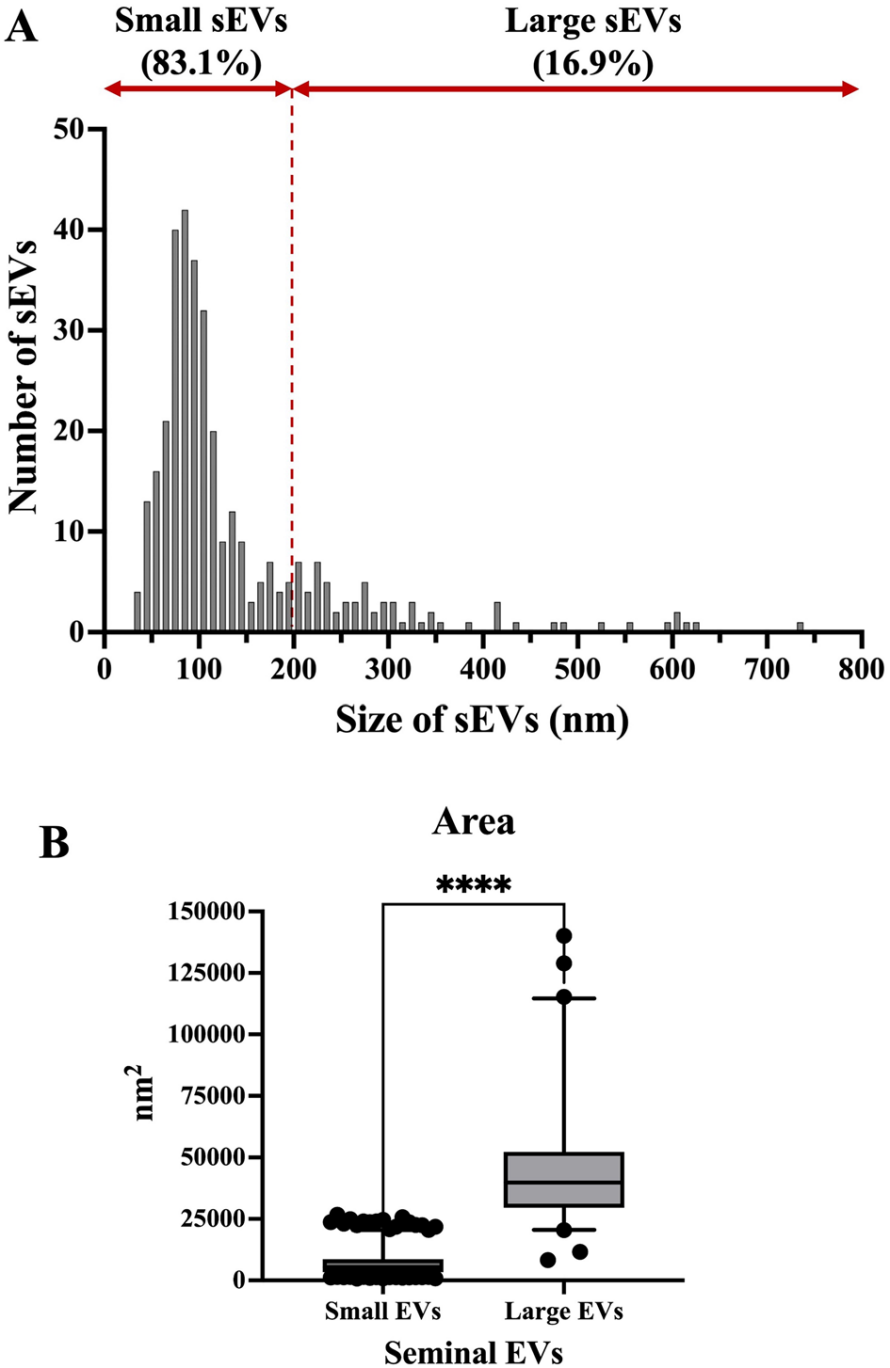
(**A**) Size distribution of extracellular particles (sEVs, n = 495) isolated from seminal plasma samples of entire porcine ejaculates. The sEVs with a maximum diameter equal to or less than 200 nm were defined as small sEVs (n = 358) and those with a maximum diameter greater than 200 nm as large sEVs (n = 73). (**B**) Boxplot showing the area of the small and large sEVs. Boxes enclose the 25th and 75th percentiles, the whiskers extend to the 5th and 95th percentiles and the line represent the median. ****P < 0.0001.

**Figure 3:**
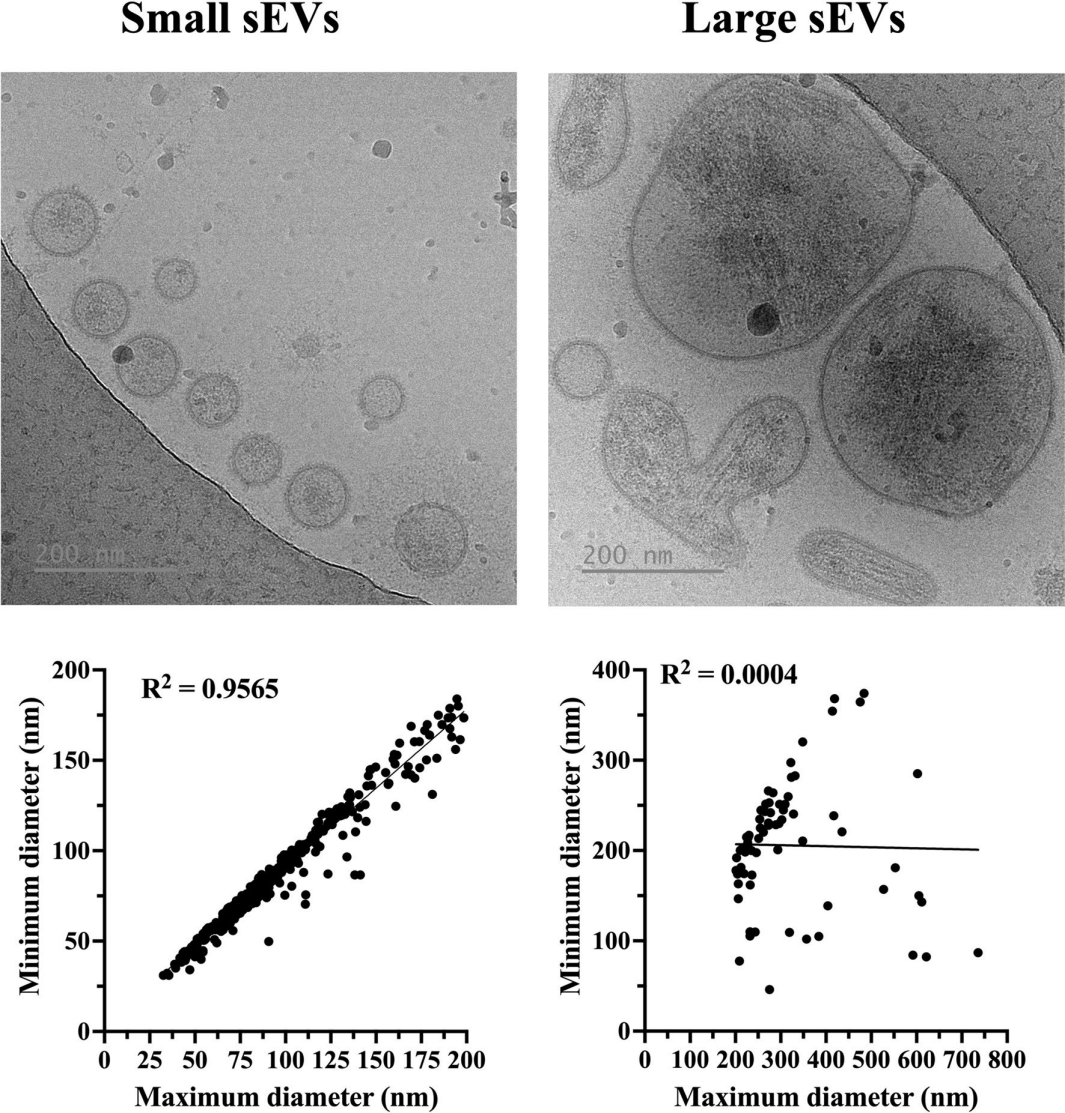
Top: Representative cryogenic electron microscopy (cryo-EM) images of small (< 200 nm in maximum diameter) and large (> 200 nm in maximum diameter) seminal extracellular vesicles (sEVs). Bottom: XY graph showing the relationship between maximum and minimum diameters in small and large sEV populations. A high R^2^ value indicates that the maximum and minimum diameters were nearly equal, indicating a rounded shape. Conversely, a low R^2^ value indicates that the maximum diameter was significantly larger than the minimum diameter, indicating oval or elongated shape.

**Figure 4:**
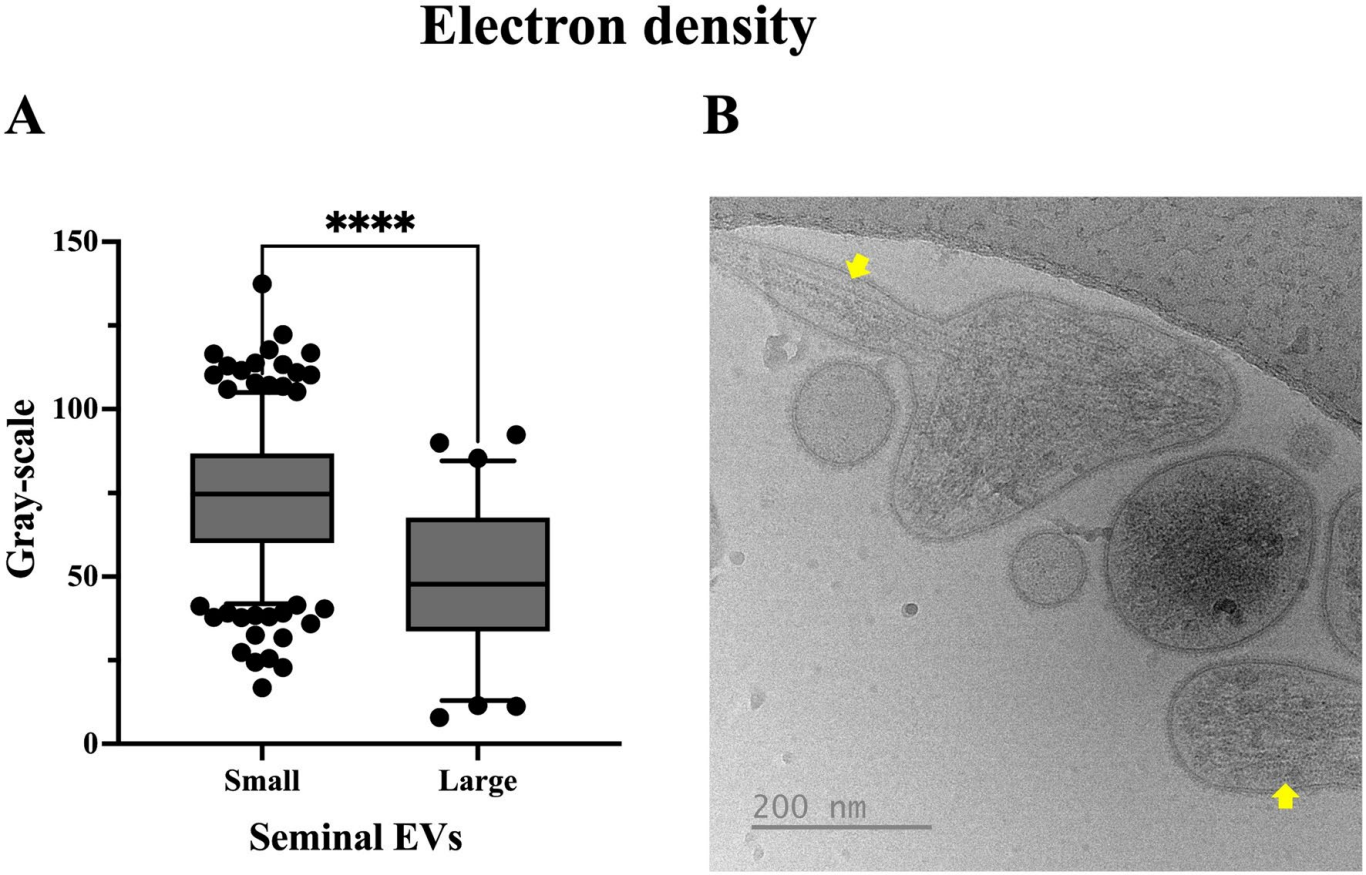
(**A**) Boxplot showing the electron density measured in a gray scale of small (< 200 nm at maximum diameter) and large (> 200 nm at maximum diameter) seminal extracellular vesicles (sEVs). Higher values in the gray scale indicate lower electron density. Boxes enclose the 25th and 75th percentiles, the whiskers extend to the 5th and 95th percentiles and the line represent the median. ****P < 0.0001. (**B**) Representative cryo-electron microscopy images illustrating differences in electron density among small and large sEVs. Yellow arrows highlight content with filamentous or filiform structures.

**Figure 5:**
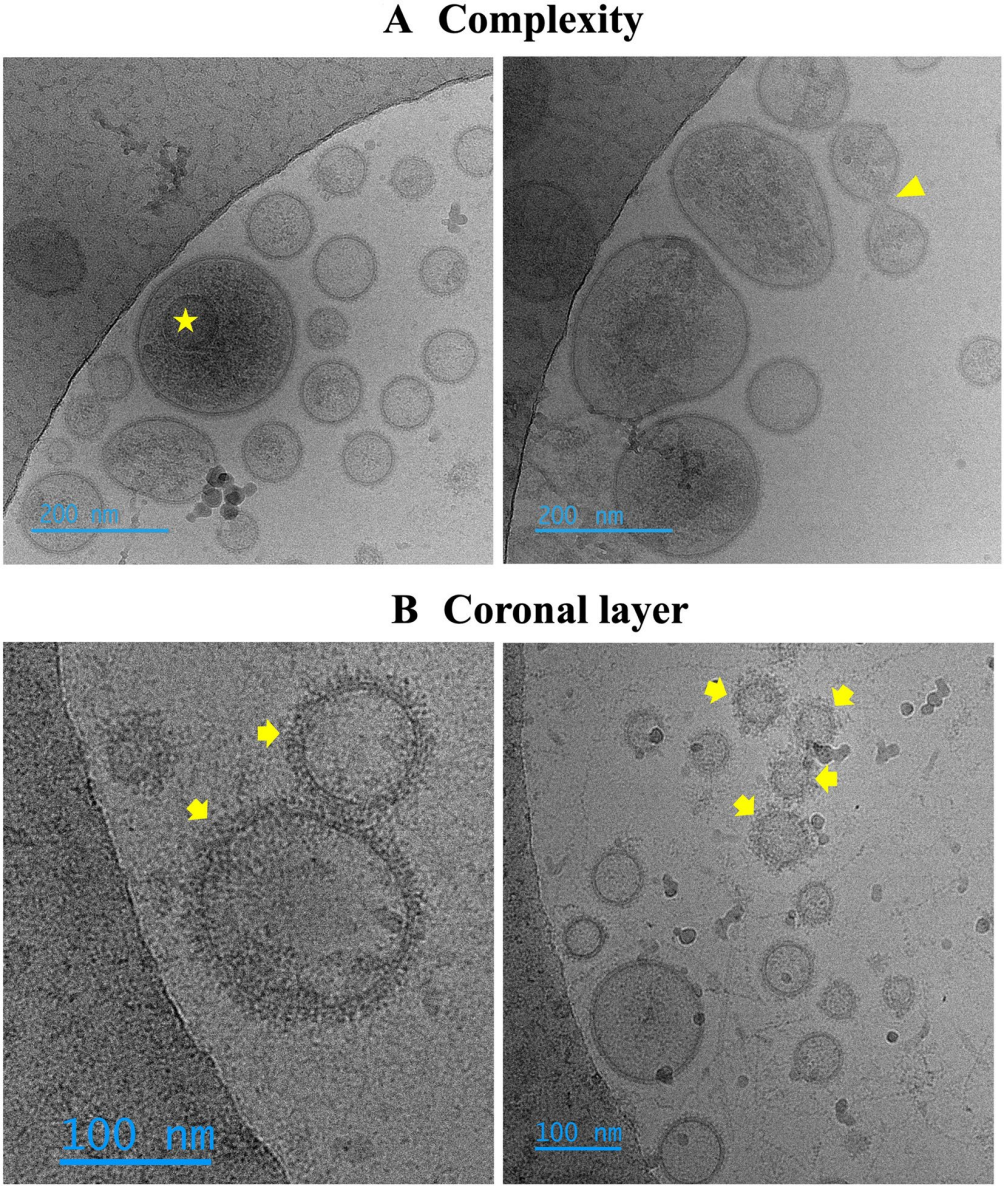
(**A**) Representative cryo-electron microscopy (cryo-EM) images showing the structural complexity of porcine seminal plasma extracellular vesicles (sEVs). Most sEVs showed simple structures and only a few had one or more small EVs within them (see sEV highlighted with a star in the left micrograph) or were connected to each other either by membrane fusion or by channels connecting the lumens (see the sEV highlighted with a triangle in the right micrograph). (**B**) Representative cryo-EM micrographs showing sEVs with a peripheral coronal layer surrounding the EV membrane. The sEVs with a visible peripheral coronal layer are highlighted by yellow arrows.

A total of 495 extracellular particles were analyzed in the sEV-enriched samples from entire ejaculates. Of these, 431 (87.1%) were bounded by a well-defined membrane and were therefore considered sEVs. The remaining 64 extracellular particles (12.9%) were not surrounded by a membrane and were therefore considered NVEPs. These NVEPs were rounded (R^2^ = 0.936), small (the maximum diameter was in the range of 24 to 46 nm), and had low electron density (108.7 ± 15.5 arbitrary units). The sEVs were heterogeneous in terms of size, shape, electron density, and presence/absence of a peripheral coronal layer (Fig. 1). The maximum diameter ranged from 33 to 736 nm, although the majority (50.4%) of the sEVs had a maximum diameter between 70 and 130 nm (Fig. 2A). The area of sEVs ranged from 793 to 140,106 nm^2^. Considering the large size variability and following the Minimal Information for Studies of Extracellular Vesicles 2023 (MISEV23) guidelines^29^, sEVs were grouped into two subsets according to their size, named small sEVs (S-sEVs, < 200 nm) and large sEVs (L-sEVs,  > 200 nm). There were more (P < 0.0001) S-sEVs (83.1%, 358/431) than L-sEVs (16.9%, 73/431) (Fig. 2A). The area of the S-sEVs (7054.5 ± 5494.2 nm^2^) was smaller (P < 0.0001) than that of the L-sEVs (45.755.6 ± 24.793.2 nm^2^) (Fig. 2B). Small sEVs were more rounded than L-sEVs (R^2^ = 0.9565 vs R^2^ = 0.0004), with ovoid and elongated shapes predominating in the L-sEVs (Fig. 3). The electron density of the L-sEVs (49.8 ± 22.2 arbitrary units) was higher (P < 0.0001) than that of the S-sEVs (74.0 ± 19.6 arbitrary units) (Fig. 4A). More L-sEVs (93.1%, 68/73) than S-sEVs (29.6%, 106/358) had visible cargo in the lumen (P < 0.001), and the cargo in L-sEVs often showed filamentous or filiform structures (67.65%, 46/68) (Fig. 4B). Regarding the complexity of sEVs (Fig. 5A), there were no differences between S-sEVs (< 200 nm) and L-sEVs (> 200 nm). Most of the sEVs showed simple structures (98.1%, 423/431), and the remaining ones (1.9%, 8/431) had one or more small EVs inside. A few sEVs (5.1%, 22/431) were linked together, either by membrane fusion or by channels interconnecting lumens. The peripheral coronal layer (Fig. 5B) was significantly (P < 0.001) more present in S-sEVs (43.1%, 154/358) than L-sEVs (15.1%, 11/73).

### Differences in cryo-EM sEV measurements among ejaculate fractions

**Figure 6:**
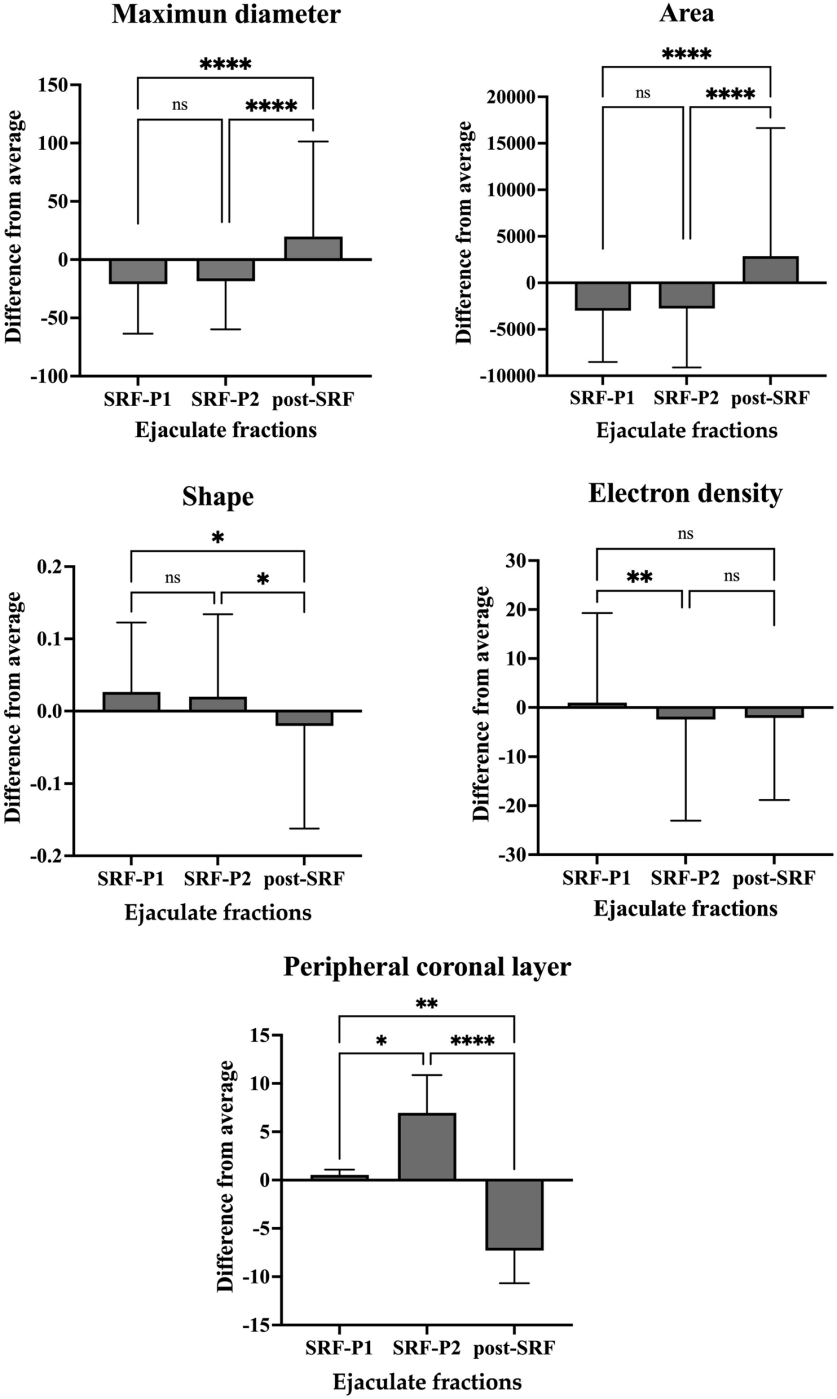
Cryo-electron microscopy measurements of porcine seminal extracellular vesicles (sEV) showing differences between the three ejaculate fractions: the first 10 mL of the sperm rich ejaculate fraction (SRF-P1), the remainder of the sperm rich ejaculate fraction (SRF-P2), and the post-sperm rich ejaculate fraction (post-SRF). Results are expressed as the difference from the mean of the entire ejaculate. The value of 0 is the mean value of the entire ejaculate, and the mean values for maximum diameter, area, shape, electron density, and presence of peripheral coronal layer were 92.30 nm, 7079.14 nm^2^, 0.89 (on a scale of 0 to 1), 109.08 (on a gray scale), and 24.78%, respectively. ****P < 0.0001, **P < 0.01, *P < 0.05. *ns* non-significant.

A total of 1409 sEVs were analyzed by cryo-EM in the SP samples of all three ejaculate fractions, divided into 742, 573 and 94 sEVs for the SRF-P1, SRF-P2 and post-SRF, respectively. The raw data and the data of the difference from the mean of the mimicked entire ejaculate were analyzed as discussed in the statistical analysis section. Statistically, both data sets showed differences between the ejaculate fractions in the same cryo-EM parameters, i.e. maximum diameter, area, shape, electron density, and presence of peripheral coronal layer. The results of these cryo-EM variables, expressed as the difference of the data set of each fraction from the mean of the mimicked whole ejaculate, are shown in Fig. 6. The sEVs from the post-SRF, the sperm-poor ejaculate fraction, were larger in diameter (P < 0.0001) and area (P < 0.001) and less rounded in shape (P < 0.05) than the sEVs from the SRF-P1 and SRF-P2, the sperm-rich ejaculate fractions. Electron density differed between the sEVs of SRF-P1 and SRF-P2, with those of SRF-P1 being less electron dense than those of SRF-P2 (P < 0.01). Finally, the peripheral coronal layer was visualized in more sEVs from SRF-P1 and SRF-P2 than from post-SRF (P < 0.0001).

## Discussion

The present cryo-EM based study demonstrated that the population of porcine sEV is structurally and morphologically heterogeneous and that some of these structural and morphological differences may be determined by the reproductive organ of origin of sEVs.

The research consists of two separate experiments, the first with sEVs isolated from SP samples of entire ejaculates, and the second with sEVs isolated from SP samples of three specific ejaculate fractions, each with a different source of SP. Focusing on the first experiment, the cryo-EM images revealed that the population of sEVs in the porcine ejaculate is structurally and morphologically very heterogeneous. This is not particularly unexpected, as the same is true for EVs circulating in any other body fluid^30,31^, including human SP^22,23^. Moreover, the heterogeneity of porcine sEV population was also demonstrated by Barranco et al.^9^ measuring EV-protein markers by flow cytometry. The heterogeneity of sEVs would be related to the diversity of sources, since sEVs are released from cells of functional organs as diverse as the testis, vas deferens, epididymis, and some sexual accessory glands, particularly the prostate and seminal vesicles. Once released, these EVs formed the heterogeneous population of sEVs present in the ejaculate^7^.

Cryo-EM allows single-EV analysis, providing insight into the structure and morphological complexity of each EV and thereby it is an excellent tool for morphologically define EV subpopulations^30^. The morphologic feature that can be most objectively measured in EVs by cryo-EM is size. According to the MISEV23 recommendation, sEVs with a maximum diameter of less than 200 nm were classified as small (S-sEVs) and those with a maximum diameter of more than 200 nm were classified as large (L-sEVs)^29^. Most of the sEVs were classified as small, and a relatively few sEVs were classified as large. Classically, small EVs were linked to exosomes and large EVs to ectosomes or microvesicles^29^. This classification is based on biogenesis pathway, and the morphological features revealed by cryo-EM did not allow to differentiate between exosomes or ectosomes. However, Bordanaba-Florit et al. in a review summarizing studies focusing on EV characterization using single vesicle technologies, concluded that the biogenesis pathway would be one of the main reasons for the existence of morphologically distinct EV populations^30^. Most of the S-sEVs were rounded, while the L-sEVs had a more heterogeneous shape. Small, round EVs would more closely resemble exosomes and larger, irregularly shaped EVs, more closely resemble ectosomes or microvesicle^19^. However, it is noteworthy that a third biogenesis pathway, apocrine secretion, has been proposed for sEVs^32^. Thus, three different subpopulations of sEVs should be defined according to the biogenesis pathways.

The electron density of the sEVs was also analyzed and objectively quantified on a gray scale, with higher gray values indicating a lower electron density. The S-sEVs were less electron dense than L-sEVs. Poliakov et al. reported differences in electron density between human sEVs^22^. In addition to a visual morphological feature, electron density would provide information about the luminal cargo of EVs^33^, and differences in electron density would indicate compositional differences, either qualitatively or quantitatively. The same hypothesis was recently proposed by Neyroud et al.^21^ to explain the differences in electron density of EVs isolated from human ovarian follicular fluid. Interestingly, our results agree with those of Neyroud et al. who also reported that large EVs were more electron dense than small EVs^21^. In some L-sEVs, which are the most electron dense, filamentous, or filiform structures were visible in the lumen. These structures may be actin-related proteins^34,35^. This assumption would be supported by the proteomic study carried out by Barranco et al. which showed a higher abundance of actin-related proteins in the L-sEVs than in the S-sEVs^36^. It is noteworthy that actin-related proteins are important for the functional performance of spermatozoa, as they play an essential role in the dynamic remodeling of the sperm cytoskeleton during capacitation^37^.

The presence or absence of the so-called peripheral coronal layer was another morphological feature evaluated, as it can be visualized with reasonable clarity using cryo-EM^24^. This peripheral coronal layer, also called the protein corona, surrounds the EV externally and is visible by cryo-EM as a thickening that coats the EV membrane. In the present study, the coronal layer was present in several sEVs, especially in S-sEVs, which would be consistent with previous studies reporting the presence of a coronal layer in small EVs^38,39^. This peripheral coronal layer is composed of molecules, mainly proteins^40,41^, although lipids and nucleic acids may also exist^42^. The peripheral coronal layer would form spontaneously by aggregation of molecules from the matrix surrounding EVs^41^. Therefore, some proteins and lipoproteins that are considered EV contaminants, such as some apolipoproteins, may be part of the coronal layer^25^. Indeed, some of these apolipoproteins that Hallal et al.^25^ associate with the coronal layer of plasma EVs were found in porcine sEVs and in higher relative amounts in S-sEVs than L-sEVs^36^. This finding would support the cryo-EM results that the peripheral coronal layer is more common in S-sEVs than in L-sEVs.

The high-resolution images provided by cryo-EM also allowed to assess the purity of the isolated EV, as all isolated particles can be clearly visualized, regardless of whether they are surrounded by a lipid bilayer or not^43^. Cryo-EM images showed that some co-isolated particles were NVEPs, i.e. non-vesicular extracellular vesicles, with a size like the smallest sEVs. In particular, the proportion of NVEPs was relatively low in all sEV samples, regardless of whether the SP was from the entire ejaculate or from a specific ejaculate fraction. This relatively low number of NVEPs suggests that the isolation procedure used, which included serial centrifugation and ultrafiltration before or after to size exclusion chromatography (SEC), allowed isolation of sEVs with an acceptable level of purity. These results would be supported by the flow cytometry analysis performed for complementary phenotypic characterization of the sEV samples, which showed a low albumin content in the sEV samples. These co-isolated NVEPs are most likely lipoproteins. Yuana et al. observed similar nanoparticles in a cryo-EM study performed on blood plasma samples and demonstrated that they were lipoproteins^15^. Lipoproteins are the main cross-contamination in samples of EVs isolated by SEC because, as mentioned above, they are similar in size to smaller EVs. Contamination by NVEPs is one of the major drawbacks of isolated EV samples, undermining the success of functional EV studies^44^. Isolation of EVs in complete purity is required but very difficult to achieve, especially for EVs from protein-rich matrices such as blood plasma or SP, and therefore remains a challenge^29^.

In the second experiment, sEVs from three different ejaculate fractions were analyzed by cryo-EM and showed some different structural and morphological characteristics. The SP of each of the three ejaculate fractions has a specific origin, i.e. the SP of the SRF-P1 comes mainly from the epididymis, that of the SRF-P2 mainly from the prostate, and that of the post-SRF mainly from the seminal vesicles^1^. Therefore, a different functional reproductive organ would be the primary source of sEVs in each of the three ejaculate fractions. The structural and morphological features that define the different EV subpopulations may not be related to their cellular origin^30^. However, depending on the organ of release, the structural and morphological characteristics of EVs may differ. At least in the case of sEVs, as demonstrated in this study. The main differences were found between the sEVs of the SRF (P1 and P2), the sperm-rich ejaculate fractions, and the sEVs of the post-SRF, the sperm-poor ejaculate fraction. The sEVs of the post-SRF were larger, less rounded, and showed less presence of the peripheral corona layer. It is not possible to predict from the present results and those reported in the literature how the structural and morphological differences of sEVs between ejaculate fractions may affect the cargo and functional roles of sEVs. However, since many of the functional roles attributed to SP would be performed by sEVs^45^, studies of the composition and functionality of SP from the three ejaculate fractions may provide some insights. Perez-Patiño et al., in a study characterizing the SP proteome of the three porcine ejaculate fractions, found a significant number of proteins that were quantitatively different between the ejaculate fractions^46^. Such differences were particularly evident between the sperm-rich ejaculate fractions (SRF-P1 and SRF-P2) and sperm-poor ejaculate fraction (post-SRF), just between the ejaculate fractions in which cryo-EM revealed the greatest structural and morphological differences in sEVs. Thus, it is possible that the quantitative protein load of the sEVs varies between ejaculate fractions. Alkmin et al. in a cryopreservation study of epididymal spermatozoa previously incubated with SP from each of the three ejaculate fractions, found that spermatozoa incubated with SP from post-SRF had poorer post-thaw survival than those incubated with SP from SRF-P1 and SRF-P2^47^. The evidenced interaction of sEVs with porcine spermatozoa^48^ may mediate this negative effect of post-SRF SP. Perez-Patiño et al. reported that post-SRF spermatozoa had poorer functional performance than those of SRF-P1 and SRF-P2, and linked this to quantitative differences in the sperm proteome between the ejaculate fractions^49^. These researchers attributed these quantitative differences in the sperm proteome between ejaculate fractions to the interaction of the spermatozoa with the corresponding SP. An effect of SP that may be mediated by sEVs. Of course, the involvement of sEVs in the effects of SP on sperm performance described in the above studies is a hypothesis and obviously needs to be verified.

In conclusion, cryo-EM analysis revealed that porcine sEVs are structurally and morphologically heterogeneous. They show clear differences in size, shape, electron density, complexity, and presence of a peripheral coronal layer. These differences would be related to the source of origin. Thus, the small sEVs, which are rounded, low in electron density, and more likely to have a peripheral coronal layer, would originate primarily from the epididymis and prostate. The large sEVs, more irregularly shaped, more electron dense, and less likely to have a peripheral coronal layer, would originate primarily from seminal vesicles.

## Methods

All animal procedures were approved by the Bioethics Committee of the University of Murcia (Murcia, Spain; CBE: 367/2020) and were performed according to the European guidelines for the protection of animals used in scientific research (Directive 2010-63-EU), and the study is presented according to the ARRIVE guidelines. The semen donors were healthy, mature (2–3 years old) and fertile Landrace × Large White boars. They were housed in an AI center under controlled environmental conditions (AIM Ibérica, Calasparra, Murcia, Spain) and subjected to regular semen collection to produce semen doses for commercial AI programs. The boars had free access to water and were fed a commercial diet designed to meet the nutritional requirements of adult boars used as semen donors. Boar management and production of AI semen doses were in accordance with Spanish (ES300130640127, August 2006) and European (ES13RS04P, July 2012) guidelines on the production and marketing of AI semen doses and animal health and welfare.

### Semen samples and SP collection and processing

A total of 21 ejaculates (one per boar) were used in the study. The ejaculates were collected during the months of February through April and all of them were suitable for the preparation of semen AI doses as they contained more than 200 × 10^6^ spermatozoa/mL with percentages of total sperm motility and normal sperm morphology greater than 75%. Entire ejaculates were collected by semi-automatic method (Collectis^®^, IMV Technologies, L’Aigle, France) and fractionated ejaculates were collected by gloved hand method. The difference in color between the SRF (milky white) and the post-SRF (translucent grayish) facilitates the fractionated collection of the ejaculates. Falcon 15 mL graduated tubes (reference 11755075, Fisher Scientific, Hampton, New Hampshire, USA) were used to collect 10 mL of each of the three ejaculate fractions: SRF-P1, SRF-P2, and post-SRF.

Immediately after ejaculate collection, SP was harvested by centrifuging 10 mL semen samples of each entire or fractionated ejaculate twice (1,500 xg for 10 min at room temperature [RT]; Rotofix 32A, Hettich Centrifuge UK, Newport Pagnell, Buckinghamshire, England, United Kingdom). The resultant SP samples were treated with a protease inhibitor cocktail (Roche complete™ Protease Inhibitor Cocktail tablets; Basel, Switzerland), stored at 5 °C, and shipped in isothermal containers to the Andrology Laboratory of the Veterinary Teaching Hospital, University of Murcia, where they were stored at − 80 °C until isolation of sEV. The 21 SP samples were stored and used for experiments. Six of them were from entire ejaculates and the other 15 were from fractionated ejaculates, five samples for each of the three ejaculate fractions, i.e., SRF-P1, SRF-P2 and post-SRF.

### Isolation of sEVs

Seminal EV samples were isolated by sequential centrifugation, ultrafiltration, and SEC, a procedure successfully applied to porcine SP by Barranco et al.^36^ The SP samples (10 mL) were thawed at 4 ºC and centrifuged at 3200 × *g* for 15 min at 4 ºC (Sorvall™ STR40, Thermo Fisher Scientific, Waltham, MA, USA) and the supernatants (2 mL) were diluted (1:2; v:v) in filtered phosphate buffer saline (PBS, 0.22 µm, Millex^®^ syringe filters) and then concentrated by ultrafiltration (Amicon^®^ Ultra-4 mL 100 kDa centrifuge filter; 3200 × *g* for 90 min at 4 °C; Sorvall™ STR40, Thermo Fisher Scientific) to a final volume of 2 mL. The 2 mL samples were subjected to SEC using homemade columns made with 10 mL of Sepharose-CL2B^®^ resin (Sigma Aldrich^®^, Merck KGaA, Darmstadt, Germany) in 20 mL SEC-tubes (Econo-Pac^®^ Chromatography Columns 20 mL, Bio-Rad Laboratories, Hercules, California, USA). The column resin was washed with 60 mL of filtered PBS and then loaded with the 2 mL samples, followed by 8 mL of filtered PBS to allow the sample to flow gently through the column (flow rate between 0.6 and 0.8 mL/min). From each SEC column, 20 eluted 500 µL fractions were collected sequentially. Based on previous studies by our research team (unpublished data), the four most sEV-enriched fractions (fractions 7–10) were selected and combined to generate a single 2 mL sEV-enriched sample from each SP sample. These sEV samples were stored at − 80 °C in an ultra-low freezer (Haier Inc., Qingdao, China) until used for phenotypic characterization.

### Cryo-EM analysis

Seminal EV samples were processed at RT for cryo-EM analysis, following the procedure adapted to the one described by Las Heras et al.^24^ and the recommendations of Cizmar & Yuana^17^. Four µL of each sEV-enriched sample were placed on glow discharged R2.1 QUANTIFOIL^®^ holey carbon film grids (Quantifoil Micro Tools GmbH, Großlöbichau, Germany). The carbon grids were placed inside the chamber of the EM GP2 Plunge Freezer (Leica), which is maintained at 8 ºC and relative humidity close to saturation (95% rH). The vitrified grids were placed in a Gatan 626 cryo-holder and imaged at − 175 °C with a JEM-2200FS/CR field emission electron microscope (JEOL, Tokyo, Japan) equipped with a 200 kV field emission gun and a cooled ULTRASCAN 4000 SP (4008 × 4008 pixels) slow scan K2 camera (Gatan Inc., Pleasanton, CA, USA). Images were acquired at magnifications ranging from 20,000x (corresponding to 0.2 nm pixel size) to 40,000x (0.11 nm pixel size) at 8 mm under focus. Cryo-EM software (DigitalMicrograph, https://www.gatan.com/products/tem-analysis/gatan-microscopy-suite-software) was used to acquire the data sets. One thousand non-overlapping images (totaling 3000 nm^2^) were acquired for each of the two experiments (entire ejaculate and fractionated ejaculate). The recorded cryo-EM images (1840) were analyzed using the open-source software ImageJ^50^. All visualized particles were recorded and those surrounded by a well-defined dark line, indicating the presence of a membrane, were considered as sEVs. Particles not surrounded by a dark line (no membrane) were also recorded and considered as NVEPs according to MISEV23 guidelines^29^. The data recorded for both sEVs and NVEPs included size, shape, electron density, complexity, and peripheral coronal layer. Size was measured as maximum diameter (nm) and area (nm^2^). Shape was recorded in terms of roundness, measured on a scale of 0–1, with values near 1 indicating greater roundness. Electron density was measured in gray scale (0–255), with higher gray values corresponding to lower electron density. The gray value of each sEV of an image was the measured gray value minus the background gray value. The background gray value of each image was the average of five measurements taken randomly at different points in the image. In addition, the presence of specific electron dense cargo within the sEVs was also recorded. Complexity was recorded as single or multiple sEVs, i.e. sEVs containing other smaller sEV within them, by recording the number inside. Finally, the presence/absence of a visible peripheral coronal layer surrounding the sEVs was recorded.

### Complementary phenotypic characterization of sEVs

In addition to cryo-EM analysis, phenotypic characterization of sEV-enriched samples was further expanded using an orthogonal approach as recommended by the MISEV2023 guidelines^29^. Orthogonal characterization of sEV samples included total protein concentration, particle size distribution, EV concentration, EV-specific protein markers, and a NVEP-specific marker. Total protein concentration was quantified using the commercial Micro BCA™ Protein Assay Kit (Thermo Fisher Scientific) on samples previously incubated with Tris-Triton lysis buffer (1:1, v:v) for 30 min at 37 °C^51^. The size distribution of sEVs was analyzed by DLS using a Zetasizer Nano ZS (Malvern Panalytical, Malvern, UK) operating at 633 nm and recording backscattered light at 173° for 150 s. Signal intensity was converted to size distribution using Dispersion Technology v.5.10 software (Malvern Panalytical), and the diameter (nm) of sEVs was calculated from the maximum peak of the Gaussian function. The sEV concentration was assessed by flow cytometry (CytoFLEX S; Beckman Coulter, Life Sciences Division Headquarters, Indianapolis, USA) as the number of particles positive for CFSE (CellTrace ™, Thermo Fisher Scientific) and expressed as sEVs per mL of sample. Identification of EV-specific protein markers and NVEP-specific marker was performed by flow cytometry according to the procedures described by Barranco et al.^52^. The EV-specific protein markers identified were the tetraspanin CD63 (anti-CD63-FITC, clone REA1055, Miltenyi Biotec, Bergisch Gladbach, Germany) and the cytosolic protein HSP90β (anti-HSP90β-PE; ADI-SPA-844PE-050, Enzo Life Sciences, Farmingdale, NY, USA). The NVEP-specific marker analyzed was albumin (anti-Albumin-FITC; CLFAG16140, Cedarlane, Burlington, Canada, USA), as it is one of the major NVEPs co-isolated with EVs^29^ and is particularly abundant in SP^46^. To ensure absence of background, autofluorescence and nonspecific antibody signal, recommended Minimum Information about a Flow Cytometry experiment and EV research (MIFlowCyt-EV) controls were performed^53^. For more details on the flow cytometry analysis procedure, see Barranco et al.^52^.

### Statistical analysis

Quantitative data were statistically analyzed using Prism software (version 10.0.0; GraphPad Software, Inc., San Diego, CA, USA). The data were first subjected to the Shapiro–Wilk test to determine whether they followed a normal distribution. The sEVs of entire ejaculate visualized by cryo-EM were divided into small sEVs (S-sEVs, < 200 nm) and large sEVs (L-sEVs, > 200 nm), and differences in size, shape, and electron density between the two sEV subsets were analyzed by Mann–Whitney test. Simple linear regression analysis was used to verify the degree roundness of sEV. Cryo-EM data from ejaculate fractions were analyzed as raw data and as deviations from the mean of data from a mimicked entire ejaculate. The cryo-EM dataset of mimicked entire ejaculate sEVs was generated by randomly pooling data from the three ejaculate fractions, with the number of sEVs from each fraction proportional to the semen volume of each fraction. Differences in size, shape, and electron density between the sEVs of the ejaculate fractions were analyzed by one-way ANOVA or Kruskal–Wallis test. Percentage data were compared using the chi-squared test. Results are presented as mean ± SD unless otherwise noted, and differences were considered statistically significant at P < 0.05.

### Supplementary Information

Supplementary Figure S1.

Supplementary Figure S2.

Supplementary Legends.

## Data Availability

Images and data supporting the findings and conclusions presented are available upon reasonable request from the corresponding author.
