# Author Correction: Cryogenic electron microscopy reveals morphologically distinct subtypes of extracellular vesicles among porcine ejaculate fractions

**DOI:** 10.1038/s41598-024-69329-z

**Published:** 2024-08-09

**Authors:** Ana Parra, Isabel Barranco, Pablo Martínez‑Díaz, Esperanza González, Oihane E. Albóniga, Diana Cabrera, Juan M. Falcón‑Pérez, Jordi Roca

**Affiliations:** 1 Department of Medicine and Animal Surgery, Veterinary Science, University of Murcia, Murcia, Spain; 2 Exosomes Laboratory, Center for Cooperative Research in Biosciences (CIC bioGUNE), Basque Research and Technology Alliance (BRTA), Derio, Vizcaya Spain; 3 Centro de Investigación Biomédica en Red de Enfermedades Hepáticas y Digestivas, Madrid, Spain; 4 Metabolomics Platform, Center for Cooperative Research in Biosciences, Basque Research and Technology Alliance, Derio, Spain; 5 IKERBASQUE, Basque Foundation for Science, Bilbao, Spain

Correction to: *Scientific Reports* 10.1038/s41598-024-67229-w, published online 13 July 2024

The original version of this Article contained errors in the Funding section.

“The study was funded by grants: PID2020-113493RB-I00/AEI/10.13039/501100011033 funded by the Spanish Ministry of Science and Innovation [MCIN], Madrid, Spain); PID2022-137738NA-I00 funded by MCIN/AEI/10.13039/501100011033/FEDER EU; RYC2021-034546-I funded by MCIN/AEI/10.13039/501100011033 and European Union (NextgenerationEU/PRTR); and 21935/PI/22 funded by the Seneca Foundation (Murcia, Spain).”

now reads:

“The study was funded by grants: PID2020-113493RB-I00/AEI/10.13039/501100011033 funded by the Spanish Ministry of Science and Innovation [MCIN], Madrid, Spain); PID2022-137738NA-I00 funded by MCIN/AEI/10.13039/501100011033/FEDER EU; RYC2021-034546-I funded by MCIN/AEI/10.13039/501100011033 and European Union (NextgenerationEU/PRTR); and 21935/PI/22 funded by the Seneca Foundation (Murcia, Spain).”

The original Article has been corrected.

