# Supplementary figures and images for: Cryogenic electron microscopy reveals morphologically distinct subtypes of extracellular vesicles among porcine ejaculate fractions
Source: Sci Rep. 2024 Jul 13;14:16175. doi: 10.1038/s41598-024-67229-w (PMC11246463; doi:10.1038/s41598-024-67229-w)

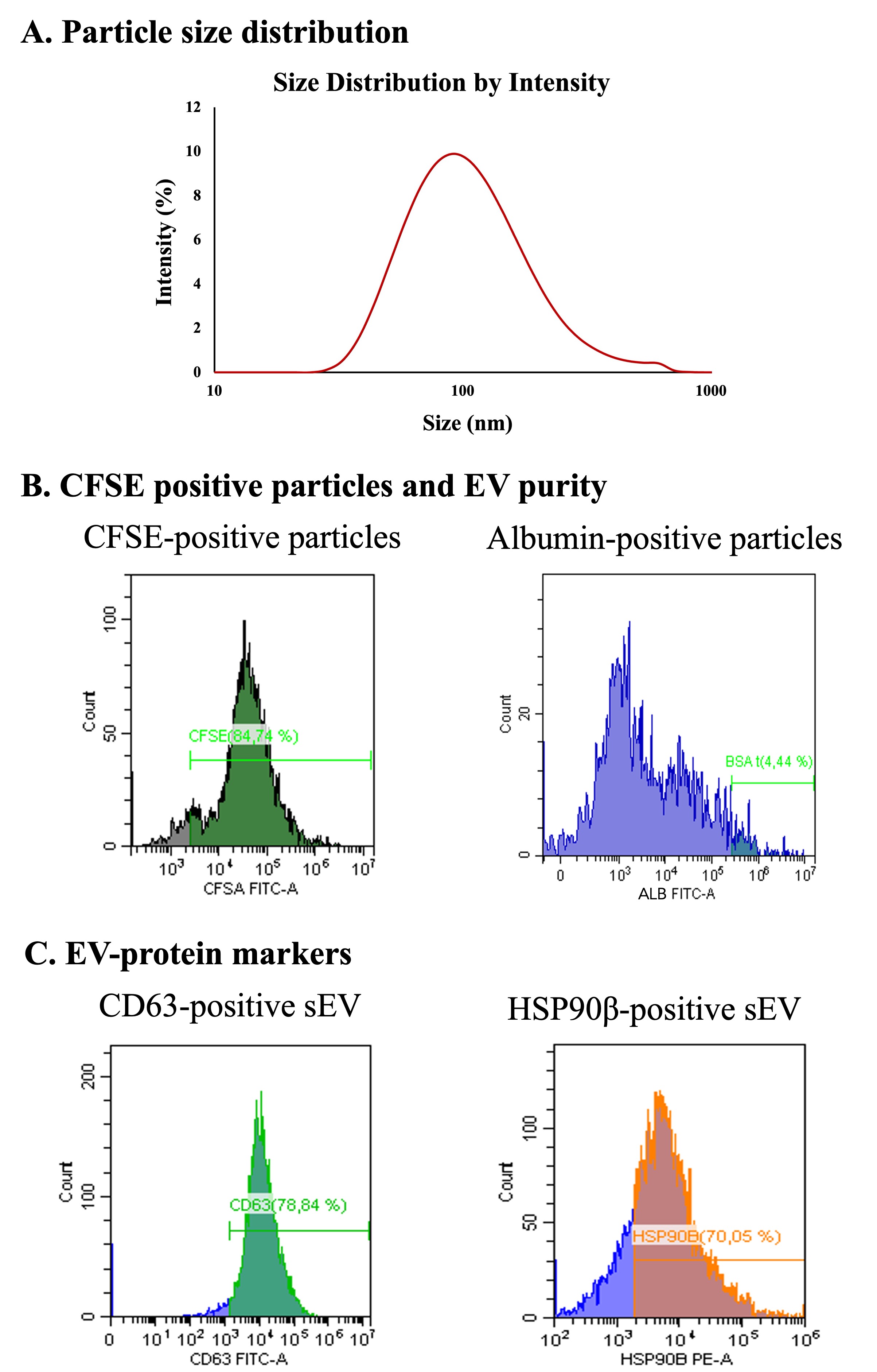

Supplement: Supplementary file 1 — Supplementary Figure S1. [file 41598_2024_67229_MOESM1_ESM.jpg]

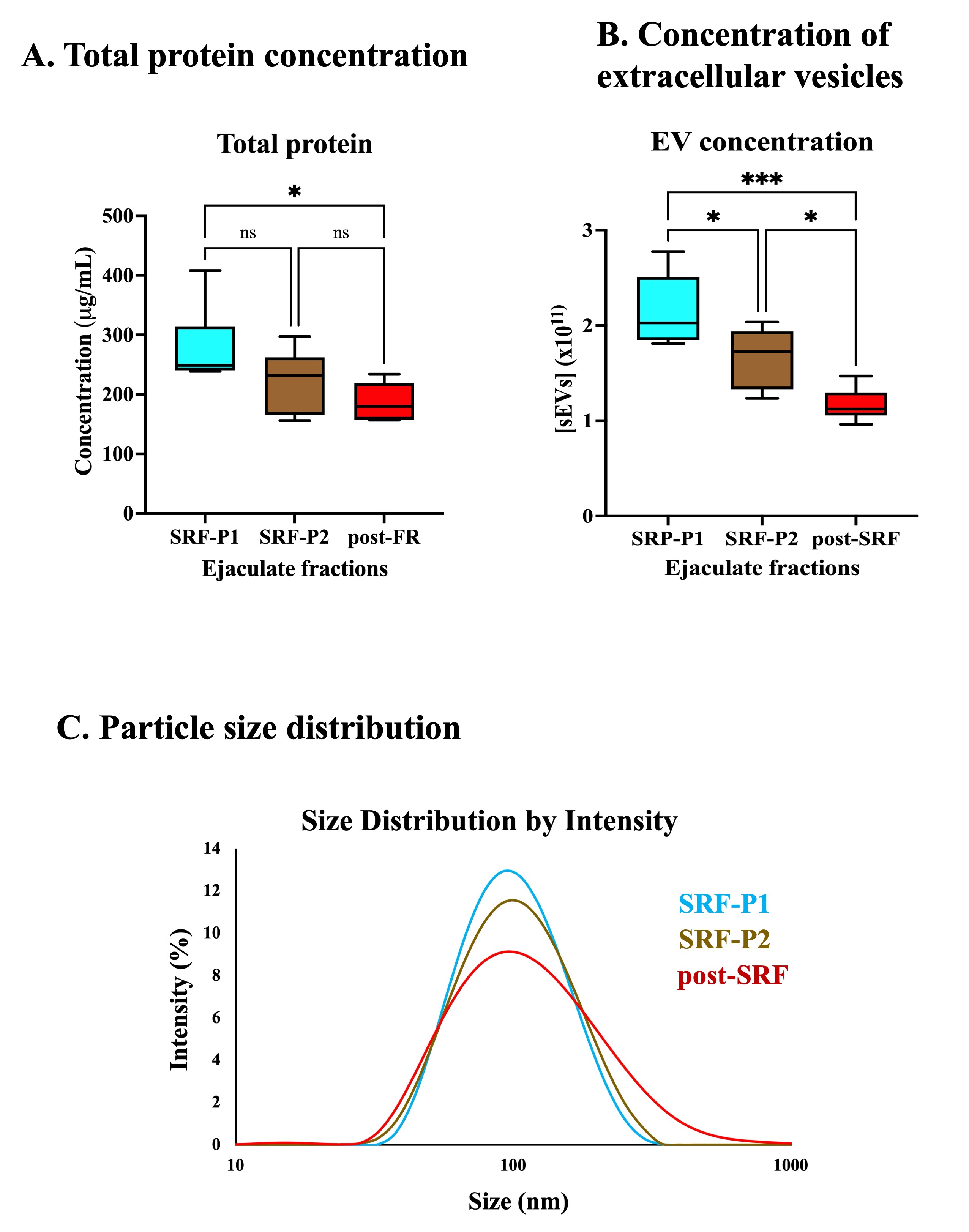

Supplement: Supplementary file 2 — Supplementary Figure S2. [file 41598_2024_67229_MOESM2_ESM.jpg]
