# Supplementary material for: Cryogenic electron microscopy reveals morphologically distinct subtypes of extracellular vesicles among porcine ejaculate fractions
Source: Sci Rep. 2024 Jul 13;14:16175. doi: 10.1038/s41598-024-67229-w (PMC11246463; doi:10.1038/s41598-024-67229-w)
Supplement: Supplementary file 3 — Supplementary Legends. [file 41598_2024_67229_MOESM3_ESM.docx]

**Supplementary figure legends**

**Supplementary Figure S1**. Phenotypic characterization of samples of porcine seminal extracellular vesicles (sEVs) isolated from entire ejaculates. (**A**) Particle size distribution as measured by dynamic light scattering. (**B**) Representative flow cytometry plots showing the percentage of particles surrounded by active membrane (carboxyfluorescein succinimidyl ester-positive particles) and the percentage of particles not surrounded by active membrane (albumin-positive particles), indicating EV purity. (**C**) Representative flow cytometry plots showing the percentage of sEVs expressing the EV-protein markers CD63 and HSP90β.

**Supplementary Figure S2**. Phenotypic characterization of samples of porcine seminal extracellular vesicles (sEVs) isolated from three specific ejaculate fractions: the first 10 mL of the sperm-rich ejaculate fraction (SRF-P1), the remainder of the sperm-rich ejaculate fraction (SRF-P2), and the post sperm-rich ejaculate fraction (post-SRF). (**A**) Total protein concentrations measured using a commercially available kit. (**B**) Extracellular vesicle concentration measured by flow cytometry (carboxyfluorescein succinimidyl ester -positive particles). (**C**) Particle size distribution measured by dynamic light scattering. ***P < 0.001, *P < 0.05, and ns (not significant).
